# Supplementary material for: Awareness-driven behavior changes can shift the shape of epidemics away from peaks and toward plateaus, shoulders, and oscillations
Source: Proc Natl Acad Sci U S A. 2020 Dec 1;117(51):32764–71. doi: 10.1073/pnas.2009911117 (PMC7768772; doi:10.1073/pnas.2009911117)
Supplement: Supplementary File [file pnas.2009911117.sapp.pdf]

Supplementary Information

Awareness-driven Behavior Changes Can Shift the Shape of Epidemics Away from Peaks and Towards Plateaus, Shoulders, and Oscillations

Joshua S. Weitz,[1,2,3] Sang Woo Park,[4] Ceyhun Eksin,[5] and Jonathan Dushoff[6,7]

[1] *School of Biological Sciences, Georgia Institute of Technology, Atlanta, GA, USA*
[2] *School of Physics, Georgia Institute of Technology, Atlanta, GA, USA*
[3] *Center for Microbial Dynamics and Infection, Georgia Institute of Technology, Atlanta, GA, USA*
[4] *Department of Ecology and Evolutionary Biology, Princeton University, Princeton, NJ, USA*
[5] *Department of Industrial and Systems Engineering, Texas A&M University, College Station, Texas, USA*
[6] *Department of Biology, McMaster University, Hamilton, ON, Canada*
[7] *DeGroote Institute for Infectious Disease Research, McMaster University, Hamilton, ON, Canada*

Typeset by REVTEX

www.pnas.org/cgi/doi/10.1073/pnas.2009911117

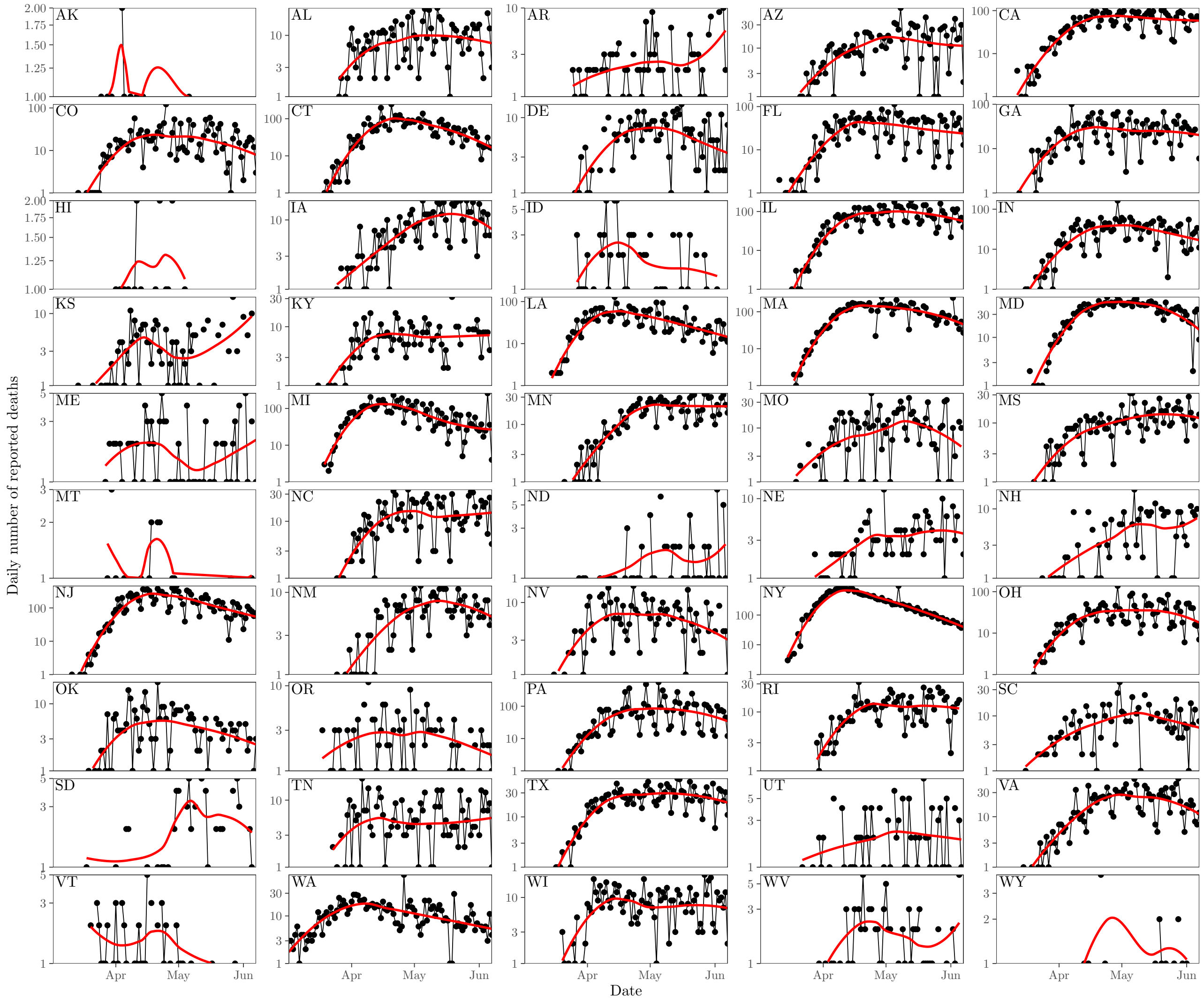

FIG. S1: Daily number of reported deaths for COVID-19 (black points and lines) and the corresponding locally estimated scatterplot smoothing (LOESS) curves (red lines) in 50 states.

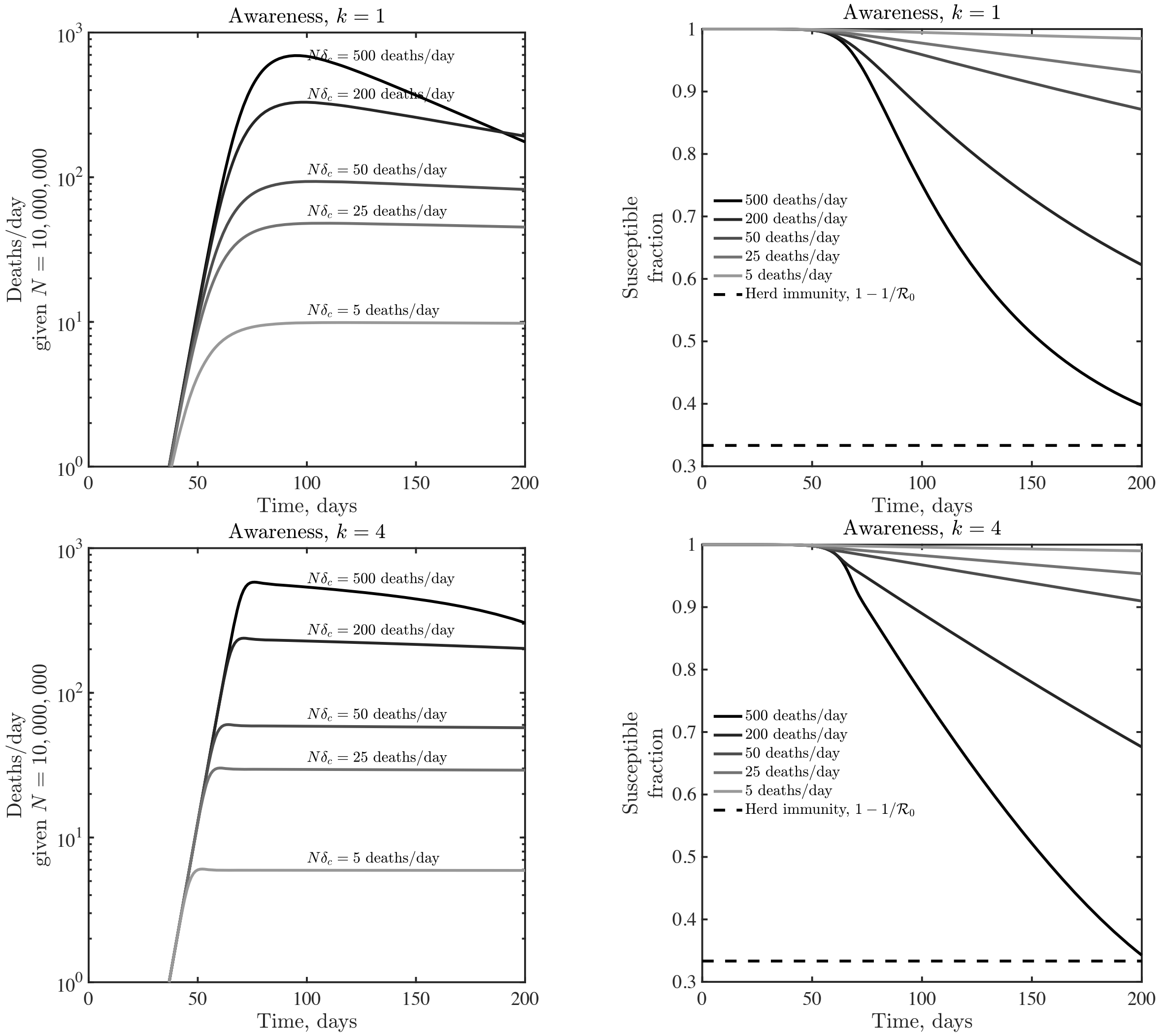

FIG. S2: Dynamics given variation in the critical fatality awareness level, δ_c for awareness $k = 1$ (top) and $k = 4$ (bottom). Panels show deaths/day (top) and the susceptible fraction as a function of time (bottom), the latter compared to a herd immunity level when only a fraction $1/\mathcal{R}_0$ remain susecptible. These simulations share the epidemiological parameters $\beta = 0.5$ /day, $\mu = 1/2$ /day, $\gamma = 1/6$ /day, and $f_D = 0.01$.

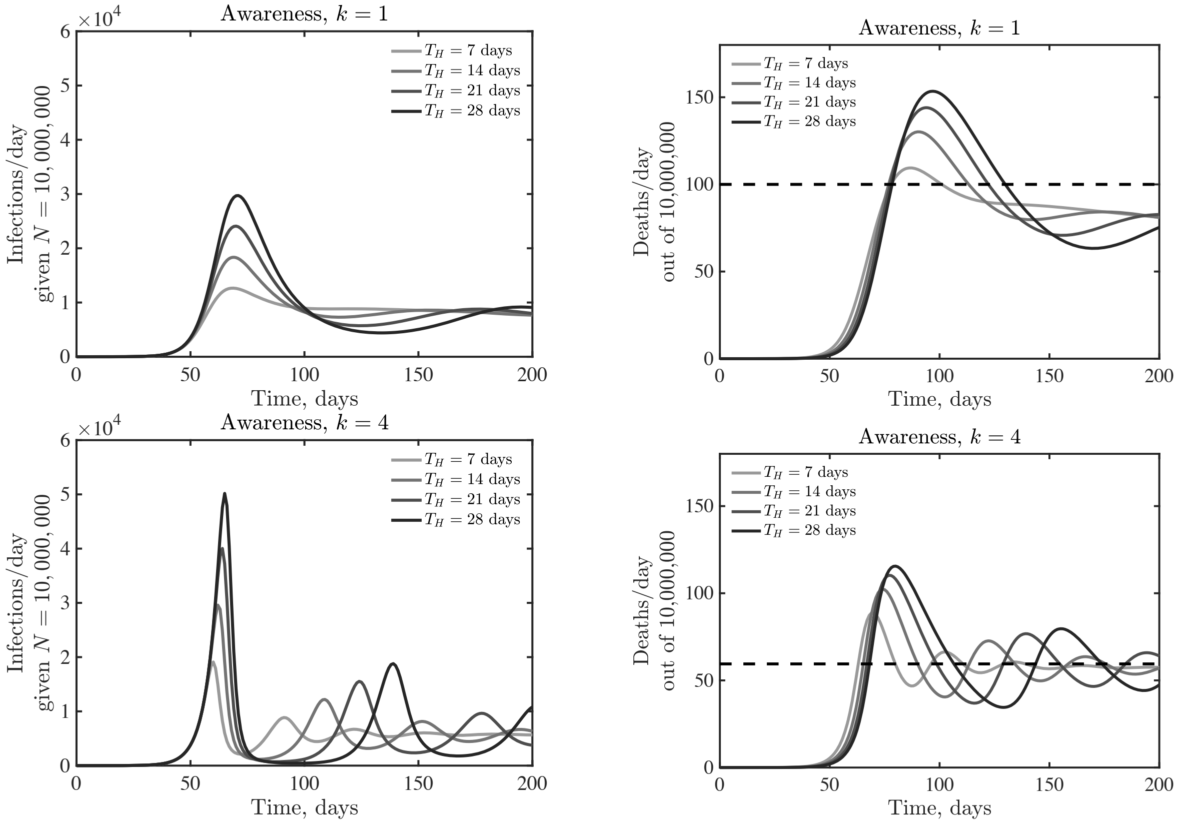

FIG. S3: Emergence of oscillatory dynamics in a death-driven awareness model of social distancing given lags between infection and fatality. Awareness is $k = 1$ (top) and $k = 4$ (bottom), all other parameters as in Figure 3. The dashed lines for fatalities expected quasi-stationary value $\delta^{(q)}$.

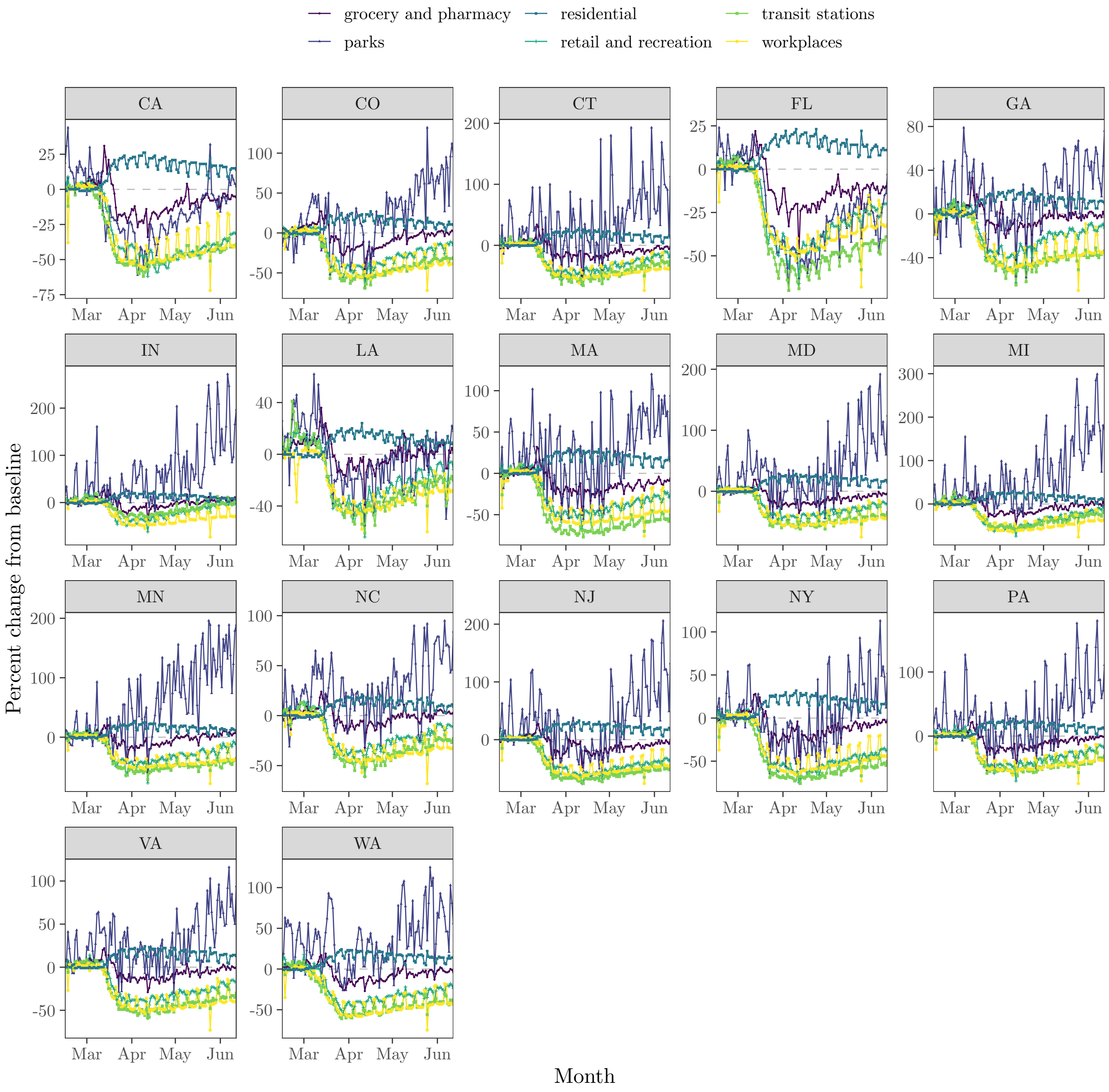

FIG. S4: Percent mobility change from baseline across six categories in 17 states.
